# Preceptors’ preparedness to teach about substance and opioid use disorder: a qualitative study

**DOI:** 10.1186/s12909-022-03922-6

**Published:** 2022-12-14

**Authors:** Enya Lowe, Alexis Coulourides Kogan, Corinne T. Feldman, Sae Byul Ma, Désirée A. Lie

**Affiliations:** 1 Division of Physician Assistant Studies, Primary Care Physician Assistant Program, Department of Family Medicine, Keck School of Medicine of USC, University of Southern California, 1000 S. Fremont Avenue Bldg #635, Alhambra, CA 91803 USA; 2 Family Medicine and Gerontology, Department of Family Medicine and Geriatrics, Keck School of Medicine of USC, University of Southern California, Alhambra, USA

**Keywords:** Health professions education, Preceptors, Clinical rotations, Opioid use, Substance use, Medication-Assisted Treatment training

## Abstract

**Study aim:**

Little is known about preceptors’ comfort and readiness to teach clinical students about the care of patients with substance and opioid use disorder (SUD/OUD). This study explores preceptors’ views about caring for such patients, and their preparedness to teach about SUD/OUD management, to improve graduate competencies.

**Methods:**

Participants were recruited by convenience and snowball sampling. Semi-structured interviews were conducted with physician, physician assistant, and nurse practitioner preceptors who taught medical and physician assistant students. Interviews were conducted via Zoom® videoconferencing. Transcripts were generated and independently analyzed for themes by 4 experienced coders using constant comparison and a grounded theory approach.

**Results:**

Fifteen interviews were conducted to theme saturation. We identified 3 major themes and 10 subthemes supported by exemplar quotes. The major themes were: education about SUD/OUD in primary care (subthemes include need for longitudinal curriculum, redefining ‘success’ in treatment, and precepting challenges), treatment of SUD/OUD in primary care (need for systemic support and care continuity), and medication-assisted therapy (MAT) training as a tool for teaching (preceptors’ own training, and need for clinical students to be trained).

**Conclusions:**

Preceptors agreed that treatment of SUD/OUD belongs in primary care and students should learn about SUD/OUD from the start of their medical education. Data analysis enabled the construction of an emerging conceptual framework reflecting a diversity of experiences and opinions of preceptor comfort and preparedness to teach about SUD/OUD, associated with various barriers and motivators. This framework can guide future strategies to address facilitators and obstacles to advance and promote preceptor preparedness to teach students about the care and management of patients with SUD/OUD.

**Supplementary Information:**

The online version contains supplementary material available at 10.1186/s12909-022-03922-6.

## Introduction

The prevalence of substance (SUD) and opioid (OUD) use disorder in the US has increased in recent years to match the prevalence of other chronic conditions such as diabetes and heart disease at 8 to 12% of the general population [1]. However, the addiction specialist provider pipeline has not grown to meet patient needs. Consequently, a shortage of mental health clinicians and specialized addiction resources for patients with SUD/OUD persists [2]. To address this service gap, recent practice guidelines [3–6] recommend that primary care providers should also participate in the detection and ongoing care of patients with SUD and OUD. Better patient access to medication-assisted therapy (MAT) is associated with improved long-term outcomes [7–9]. So, in April 2021, the US Department of Health and Human Services waived the training requirement for 8 to 24 h of MAT training [10] to allow physicians, physician assistants (PAs), and nurse practitioners (NPs) to prescribe buprenorphine for up to 30 practice patients [11]. This policy is a helpful initial step to address the barrier to MAT prescribing in primary care. However, more action is needed to ensure that future generations of providers are well-qualified to care for patients with SUD/OUD.

Educators, in particular preceptors, play key roles in preparing and improving the competency of future providers. At present, little is known about whether preceptor SUD/OUD training may be needed to enhance clinical teaching for students. Literature on teaching students about patients with addiction suggests students harbor negative attitudes or bias toward patients with addiction in contrast to those with other chronic diseases [12]. But students who received dedicated pre-clinical addiction medicine training (e.g. through lectures, case-based learning, patient narratives) were found to have a more positive outlook on addiction management [13]. Understanding preceptors’ attitudes about the care of patients with SUD/OUD and perceived value of teaching this is a prerequisite to developing appropriate tools to optimize student learning.

We therefore sought to learn about current and future preceptor attitudes and opinions through semi-structured interviews. Our study aimed to characterize preceptors’ comfort level with caring for patients with SUD/OUD and gather opinions about how students should learn about SUD/OUD during clinical rotations. The research questions were: 1. What are current and future preceptors’ perceptions about caring for patients with SUD/OUD? and 2. How prepared and comfortable are preceptors to teach students about the management of SUD/OUD?

## Methods

This study was conducted at a university-based 33-month Master of Physician Assistant (PA) Practice program in Los Angeles, California. The Institutional Review Board of the University of Southern California approved the study. The COnsolidated criteria [14] for REporting Qualitative research (COREQ) informed the study method.

### Participants and procedures

Our goal was to elicit a broad range of opinions reflecting preceptors’ clinical and teaching experiences. Thus, two types of participants were recruited: current (experienced) preceptors and future (new) preceptors. Current preceptors were actively teaching clinicians (i.e., physicians, physician assistants and nurse practitioners) with the Keck School of Medicine Primary Care PA program. Future preceptors were recent PA graduates of our program who graduated between 2017 and 2019 and planned to become preceptors. This was a convenience sample as we had access to the graduates. We targeted current primary care preceptors from Family and Internal Medicine because these are the settings in which the new SUD/OUD practice guidelines are most relevant and where our students spend most of their rotations. We also recruited subspecialty providers from Addiction Medicine, Emergency Medicine, Orthopedics, and Mental Health for added breadth of opinions.

First, participants were recruited from a convenience sample of future (*n* = 154) and current (*n*= 125) program preceptors who had been invited to an online survey on MAT training (specifically, relevance and importance of MAT training in PA education, the role and responsibility of primary care clinicians in OUD/SUD, interest in related educational topics, confidence in employing various strategies in the management of patients with SUD, perceived barriers) from January to March 2021. This survey contained a question field that allowed participants to volunteer for an interview by submitting their email address. Our rationale was that survey respondents who are preceptors would have an inherent interest in and opinion about the research question [15]. Second, we used snowball sampling among interview participants to identify other colleagues who could be referred to participate [16]. Participants were provided a $40 Visa e-gift card in appreciation of their time.

### Question guide

Interviews were guided by a semi-structured question guide informed by the literature review [11, 17–22] and developed by the study team (EL, ACK, CF, SM, and DL). The study team comprised researchers with diverse qualitative expertise in medical and PA education and educational research, clinical practice, pharmacy, health systems research, person-centered care, substance abuse and anthropology. Our research question focused on participants’ perceptions about caring for patients with SUD/OUD and their preparedness for teaching clinical students about clinical care and management of SUD/OUD. Of four open-ended questions, one focused on patient care and one on teaching, while a third covered both. The fourth question addressed opinions about MAT training. The 4-question guide was pilot-tested with an experienced NP preceptor to assess clarity and appropriateness of the questions and probes, then revised based on their feedback (See Table 1). A set of 13 brief demographic and clinical practice items was collected at the end of each interview to allow the research team to characterize the study sample (see Table 2 for demographic data).

**Table 1:** In-depth Interview Question Guide for Learning about Preceptor Perceptions about Patients, University of Southern California Primary Care Physician Assistant Program, 2021

Key Questions	Probes
1. What is your opinion about the importance of caring for patients with SUD and OUD in your practice?	- What are your thoughts on primary care providers managing substance abuse disorder or opioid abuse disorder? - What is a typical process for you when caring for a patient with SUD/OUD?
2. How important is it to you to teach about patients with SUD and OUD in your practice?	- How would you create opportunities to teach about this topic? - How do patients with SUD or OUD respond to having a student in your clinic? - What are some barriers to teaching students about these patients? - What do preceptors need to enhance this teaching in clinics?
3. Tell us about your experience caring for patients with SUD and OUD. How does this experience contribute to or hinder your teaching students about the conditions?	- How often do you care for these patients, and how much of your practice does it take up? - How is teaching about SUD and OUD in your clinic different from teaching about other conditions like diabetes or women's health or STDs?'
4. MAT waiver is no longer required to prescribe to fewer than 30 patients, but we are still considering requiring MAT waiver training for PA students before graduation. What do you think about this?	- What are the pros and cons of students getting MAT waivered before graduation?

**Table 2:** Current and Future Preceptor Interviewees, Participant Characteristics (*n* = 15), University of Southern California Primary Care Physician Assistant Program, 2021

	Frequency (%)		
	Current Preceptors	Future Preceptors	Overall
	(*n* = 10)	(*n* = 5)	(*n* = 15)
Age (mean ± SD)	44.4 ± 6.4	31.2 ± 3.6	39.7 ± 8.5
Years in practice (mean ± SD)	9.98 ± 4.3	2.8 ± 1.1	7.3 ± 4.9
Years precepting health professions students (mean ± SD)	9.0 ± 4.2	1.2 ± 1.6	6.4 ± 5.2
Racial identity (*n* = 15)			
White	5 (50.0)	2 (40.0)	7 (46.6)
Asian	1 (10.0)	1 (20.0)	2 (13.3)
African-American	1 (10.0)	0 (0.0)	1 (6.67)
Other	2 (20.0)	2 (40.0)	4 (26.0)
Prefer not to disclose	1 (10.0)	0 (0.0)	1 (6.67)
Gender identity (*n* = 15)			
Male	4 (40.0)	4 (80.0)	8 (53.3)
Female	5 (50.0)	1 (20.0)	6 (40.0)
Non-binary	0 (0.0)	0 (0.0)	0 (0.0)
Not listed	0 (0.0)	0 (0.0)	0 (0.0)
Prefer not to say	1 (10.0)	0 (0.0)	1 (6.67)
Profession (*n* = 15)			
MD	8 (80.0)	0 (0.0)	8 (53.3)
PA	1 (10.0)	5 (100.0)	6 (40.0)
NP	1 (10.0)	0 (0.0)	1 (6.67)
Multi or Single Specialty (*n* = 15)			
Single specialty	4 (40.0)	5 (100.0)	9 (60.0)
Multispecialty	6 (60.0)	0 (0.0)	6 (40.0)
Estimate on an annual basis, how many students will you work with? (*n* = 14)^a^			
None	0 (0.0)	3 (60.0)	3 (21.4)
1–9 students	1 (11.1)	2 (40.0)	3 (21.4)
10–19 students	3 (33.3)	0 (0.0)	3 (21.4)
20–29 students	2 (22.2)	0 (0.0)	2 (14.3)
30–39 students	1 (11.1)	0 (0.0)	1 (7.1)
40–49 students	0 (0.0)	0 (0.0)	0 (0.0)
50 or more students	2 (22.2)	0 (0.0)	2 (14.3)
No	1 (10.0)	0 (0.0)	1 (6.67)
How many students have you worked with in the past per year? (*n* = 15)			
None	1 (10.0)	4 (80.0)	5 (33.3)
1–9 students	2 (20.0)	0 (0.0)	2 (13.3)
10–20 students	6 (60.0)	1 (20.0)	7 (46.7)
More than 20 students	1 (10.0)	0 (0.0)	1 (6.7)
Born in USA (*n* = 15)			
Yes	9 (90.0)	5 (100.0)	14 (93.3)
No	1 (10.0)	0 (0.0)	1 (6.67)
Completed MAT training (*n* = 15)			
Yes	8 (80.0)	4 (80.0)	12 (80.0)
No	2 (20.0)	1 (20.0)	3 (20.0)
Specialty			
1 – Primary Care	8 (80.0)	1 (20.0)	8 (53.3)
2 – Subspecialty Care	2 (20.0)	4 (80.0)	7 (46.6)

^a^one response = 'unsure' due to COVID

### Data collection and analysis

Our goal was to learn about the ‘why’ and ‘how’ of preceptors’ attitudes and opinions on teaching clinical students about SUD/OUD. We chose to engage participants in interviews instead of focus groups to protect participant privacy and increase sharing of sensitive personal views [23, 24]. Interviews were conducted between March and June 2021 via Zoom® videoconferencing as in-person interviews were not feasible during the Covid-19 pandemic. Zoom® conferencing allows for face-to-face interaction and mimics in-person interviewing. Several studies [25–28] have affirmed the veracity and validity of videoconferencing compared with in-person interviewing for data collection. All interviews were conducted by an experienced anthropologist and qualitative researcher (EL) who did not have a prior relationship with the participants. Quality control was monitored by a second expert qualitative researcher (DL) who provided observed real-time feedback and transcript review on three interviews. Interviews were audio-recorded, automatically transcribed by Zoom® software, then reviewed for accuracy by the interviewer. An audit trail of the process was maintained using notes and team meeting minutes from recorded Zoom® meetings. The COREQ checklist [14] of criteria was followed and applied throughout the project.

Our thematic analysis approach was primarily *semantic* and *inductive *[29] in that we sought to not only use the raw narrative data to group ideas but also interpreted deeper preceptor meanings as they added their own opinions about patient care in response to questions about education. Concurrent with data collection, transcripts were independently read and coded by three research team members (EL, DL, and ACK) using constant comparison to identify major themes and subthemes. A fourth coder (CF) participated as an adjudicator to resolve disagreements. A code book was developed at an initial coding meeting to extract major themes and subthemes from the first three transcripts. Themes and subthemes were modified, consolidated, and sorted by all four coders during four subsequent coding meetings until 100% consensus was reached. Themes were thus derived directly from participant data using a modified grounded theory approach [30–32].

## Results

### Participant characteristics

Twenty-one preceptors from the online survey indicated their interest to participate in a Zoom® interview, and half (*n* = 11, 52%) of the initial twenty-one completed an interview. Ten participants did not respond to follow-up emails. Four additional participants were recruited via snowball sampling, resulting in a total of 15 interviews. Participants comprised current (*n* = 10) and future (*n* = 5) preceptors. Nine represented primary care (5 MDs, 2 PAs, 1 NP), six were from subspecialties (2 MD, 4 PAs). See Table 2 for participant demographics. Out of the 15 preceptors, 13 (9 current, 4 future) were MAT-certified. Interviews lasted on average 35 to 45 min.

### Themes

Thematic saturation was achieved after 11 interviews. Four additional interviews were conducted for confirmation. The codebook created by the three primary coders after analysis of the first 3 transcripts yielded three major themes and ten subthemes. Subsequently the three primary coders generated a further 4 major themes and 4 subthemes. Discussion over 4 coding meetings with adjudication by the 4^th^ coder finally resulted in a total of 3 non-overlapping major themes with 2 to 3 subthemes each. While our research question focused on clinical teaching, we found that preceptors were eager to share their approach to patient management.

The 3 major themes (see Table 3) were: education about SUD/OUD in primary care, treatment of SUD/OUD in primary care, and MAT training as a tool for teaching about SUD/OUD in primary care. Overall, we found no differences in opinion expressed by profession (MD, NP or PA), specialty (primary vs subspecialty care) or other demographic factors. Some differences were noted between current and new preceptors. Themes are further discussed with their corresponding subthemes below.

**Table 3:** Major Themes, Subthemes and Selected Quotes, Attitudes Toward Teaching about Substance and Opioid Disorders, University of Southern California Primary Care Physician Assistant Program, 2021

Major Themes	Sub Themes	Specialty	Typical Quotes	ID
Education	Enhanced, integrated longitudinal	**IM**	‘learning compassion and management is something you learn on the job being in the space having a preceptor guide you when you're being exposed.’	**203**
		**FM**	‘…students experiencing through the eyes of the patient allows them to be in that person's shoes … and takes away judgment …that's important when caring for patients with addiction.….’	**215**
		**FM**	‘(SUD/OUD education) needs to be longitudinally integrated at every level. First year, students should get addiction and understanding physiologically. The way they learn is with repetition … then the topic should be integrated into clinical.’	**211**
	Redefining success in care of SUD/OUD patients	**FM**	‘Maybe the patient is not able to give up heroin or fentanyl, but they made sure that there is Narcan around. Preceptors can help students understand that we measure success differently than what they are used to. Preceptors can show students that (the patient) didn't use this morning, or they haven't had any abscesses. Helping people to understand success at different levels is important’	**213**
	SUD/OUD presents challenges when precepting	**FM**	‘the biggest barrier (is) I don't have enough (patients with SUD/OUD). If you don't see something often it's hard to build your knowledge base because one case is one case and the next case is totally different… just seeing one or two patients doesn't give that broad overview of different stages and complexity.’	**214**
Treatment in primary care	Site integration and systemic systematic support	**FM**	‘it is shifting the culture, shifting mindset that takes years… it has to be concerted effort, not just from like the clinical leadership but operational leadership too.’	**211**
	Treatment continuity in primary care	**MH**	‘Setting the foundation for successful treatment: that's primary care. We don't need to have patients be in a specialized facility to get them started… or getting connected with behavior health. The more we shift our mindset towards (primary care), it's going to be best.’	**210**
MAT waiver as teaching instrument	Prepares providers	**FM**	‘Everyone should be trained in (MAT waiver) You're not doing a service to your community, medical students or residents, or your patients (without this training).’	**208**
	Improves employment prospects	**AM**	(MAT waiver training) makes you more competitive. It's a growing issue in primary care; if you know if you want to work in psych or addiction medicine it definitely makes you more competitive as a provider.'	**205**
	Barriers	**FM**	‘…I had a hard time finding somebody that was X-waivered that could be my mentor.’	**201**
		**MH**	“24 h (of training for MAT waiver) is too much time … we focus so much on suboxone but you're also going to see alcohol and meth depending on your setting, or smoking.’	**210**

Index: *FM* Family medicine, *IM* Internal medicine, *AM* Addiction medicine, *MH* Mental health

### Education about SUD/OUD in primary care

This theme reflected opinions about education, with 3 subthemes covering the importance of teaching and how education should be incorporated into health professions training. There was a strong positive opinion expressed by all preceptors about the importance of including SUD/OUD in medical or PA education. The first subtheme was the need for *enhanced, integrated longitudinal curricula* so that the topic of SUD/OUD is frequently addressed throughout training. Preceptors suggested that this approach would convey the importance and relevance of SUD/OUD in all patient care. For example, one current preceptor said:

*‘…(students) should be learning about (SUD/OUD) from day one, from didactic through clinical rotations through residency. It's everywhere, no one will escape seeing patients with opioid use, no matter what field … the training should reflect that.’ (T8).*

Two important findings emerged under the first subtheme. The first was the importance of *role modeling empathy* and *avoiding stigmatization* of patients as an integral and important part of teaching students. Preceptors explained that subconscious behaviors by preceptors—both positive and negative—set an example for students, which can impact students’ future behaviors with patient. For example, one current preceptor noted:

*‘With learners, you always must start with acknowledging our biases, especially with SUD, (when) there's so much stigma in society. And in medicine, we hold on to these biases even in the language we use ... It's why calling someone an ‘addict’ can be problematic and not helping build that trust and rapport’ ... Teaching the language (to students) is super important. (T11)*

The second linked preceptors’ own personal views of SUD/OUD with their *observable behaviors during patient care and teaching*. Some expressed the view that SUD/OUD as a condition should be normalized as part of chronic disease care. One current preceptor pointed out the following:

*‘SUD/OUD should be addressed the same way (as other diseases), not something that the patient brought on himself... we should be collaboratively working with the patient to try to improve on this disease.…’ (T9)*

The second subtheme reflected the role of *redefining the concept of treatment ‘success’* for patients with SUD/OUD and compared SUD/OUD care with other chronic diseases. Preceptors explained that success did not look the same for each patient and that helping students understand this is key to preparing them for treating patients with SUD/OUD in the future. One future preceptor offered this idea:

*‘Like hypertension, diabetes it’s like you have your ups and downs sometimes you’re stable sometimes you’re controlled sometimes you’re not controlled. So, if you look at addiction through that same filter it helps you understand it more and be more empathetic with the patients.’ (T5)*

In the final, third subtheme, preceptors highlighted *challenges* they face when precepting students in general, and implications for SUD/OUD training, specifically. For example, preceptors noted challenges resulting from high patient volume, time constraints, and topic sensitivity. Preceptors explained that systems challenges (e.g., lack of mentorship, lack of system resources, lack of length of time to counsel) impacted time available to address SUD/OUD in their precepting. This subtheme best reflected a spectrum of comfort levels with teaching about SUD/OUD, ranging from highly comfortable among preceptors who cared for such patients to uncomfortable and unprepared for those not MAT waivered or with low SUD/OUD patient volume. One future preceptor commented:

‘…*it is a little tricky because ... it is a little bit of a higher sensitivity … if I were to have a conversation about addressing (a patient’s) addiction with a student I might feel even a little uncomfortable*.’ (T2)

### Treatment of SUD/OUD belongs in primary care

Most preceptors believed that the treatment of SUD/OUD belonged in primary care. This opinion was qualified by perceived limitations of primary care, which included lack of systemic support to care for patients with SUD/OUD. One subtheme was the importance of the *integration of resources to best support* their care of patients. The availability of such support undergirds their ability to effectively teach students. Preceptors explained that inadequate resources most commonly resulted in an unnecessary patient referral to specialists. As well, inadequate support for their own clinical care of patients (expressed in particular by current preceptors) was associated with lower comfort and preparedness to teach students. Current preceptors felt that students were in a unique position to help mitigate the lack of clinician time because students had more time to spend with patients and to locate further supportive resources. For instance, a current preceptor mentioned:

*‘…we just didn't have a lot of resources to help the patients with. I always encourage students to delve into the deeper issues with patients… They need to do the screening for use and see how it's affecting (the patient) and do the counseling…. it's a really important role that students can play … they (students) don't necessarily know what to do, but they know what to say.” (T15).*

*Continuity of care as the* key to effective treatment formed the second subtheme. Preceptors recognized the unique position of primary care in providing continuity of services and holistic care for individual patients. One current preceptor remarked:

*‘SUD and OUD should be part of primary care bread and butter. It's what we do day to day, not a specialized topic... destigmatizing (SUD/OUD) is important and all of us should feel comfortable with it if we want to deliver whole person care.’ (T11)*

### MAT training is a tool for teaching about SUD/OUD in primary care

This theme focused on utilizing MAT training as a tool for teaching about SUD/ OUD in primary care. Preceptors who had completed MAT training indicated that they found the information useful and informative, and the waiver was valuable for practice. The majority supported required MAT training for students before graduation. Two felt the training should not be a requirement for graduation. Their reasoning was that the information was too narrow and focused only on the use of buprenorphine to the exclusion of other substances and medications. The first subtheme stressed the importance of MAT training to *prepare students for future patient care*. Waivered current preceptors were in support of required MAT training for students, whereas non-waivered current preceptors disagreed with requiring students to complete MAT training. Future preceptors, regardless of their current MAT waiver status, wished that their programs had offered the training during their own professional training. One future preceptor stated:

*‘...definitely a great thing to have as part of our (required) curriculum, just like how we get a BCLS (Basic Cardiac Life Support) trained in that kind of thing... if you don't end up prescribing buprenorphine or using it, I think it's still beneficial’ (T2)*

In the second subtheme, future preceptors, in particular, noted that MAT training was an advantage for seeking employment as the qualification set them apart from other candidates. For example, a future preceptor noted:

*‘There aren't a lot of people doing it, so you have a market edge. It makes you more marketable.’ (T8)*

Potential *barriers for prescribing* formed the third subtheme. Mentorship and time were identified as significant barriers. Both current and future waivered preceptors noted they had a difficult time finding practice mentors which limited their ability to apply or teach the skill. Time to complete MAT training was also a barrier. A current preceptor explained it as such:

*‘I think both 8 and 24 hours (of training) are way too many hours. We don't have that for any other medication. A different way of doing things would just be to have a shorter (training) …’ (T13)*

## Discussion

**Fig. 1:**
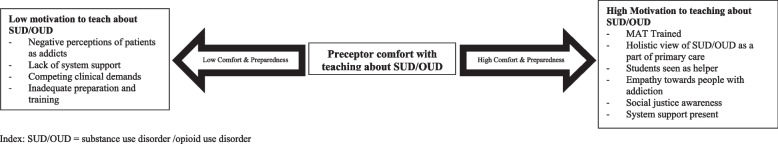
Emerging Conceptual Framework for the Preceptor Comfort and Preparedness with Teaching about SUD/OUD, University of Southern California Primary Care Physician Assistant Program, 2021

We conducted a semi-structured interview study to explore and examine current and future preceptors’ attitudes about teaching students on clinical rotation about SUD/OUD management. We discovered a diversity of experiences and opinions of comfort levels among preceptors, underpinned by factors best characterized as barriers and motivators. Our data can be expressed using the Emerging Conceptual Framework presented in Fig. 1. Preceptors, both current and future, uniformly expressed that competence in the care of SUD/OUD is a priority in health professions education because of the ubiquity of the condition and the need for primary care providers to take a frontline role. In alignment with prior studies, our preceptors advocated for a longitudinal, integrated curriculum in SUD/OUD [22, 33–35] for students. The themes that emerged also suggest that preceptors recognize significant barriers to teaching. Our study affirms negative attitudes toward patients with addiction as a barrier to care, which may be exacerbated by inadequate systemic support including mentors for prescribing, and heavy patient loads with time constraints. This limits the time available for preceptors to fully manage and teach young learners about patients with SUD/OUD. On the other hand, preceptors identified positive motivators to teach their students. Notably, preceptors who had the availability of mentorship and local support were enthusiastic about training clinical students. This substantiates the need to address systemic barriers that can discourage clinicians from caring for and teaching about this population.

Our finding that providers demonstrate a spectrum of attitudes (from negative or judgmental to supportive) toward patients with addiction has been reported in other studies [36–38]. Our study is unique in further exploring the impact these attitudes have on student learning. Preceptors in our study were aware that unconscious and conscious biases must be addressed and empathy for patients with SUD/OUD was learned through reflection and mindful positive role-modeling by preceptors [18, 21, 37]. This suggests that unconscious bias training for preceptors and future providers should include people with addiction and is likely to influence effectiveness of teaching [39, 40]. Similar to documented reports about preceptor attitudes toward patients with alcoholism and other addictions, our participants noted that more contact with patients with addiction would elicit more positive attitudes about their treatment [41, 42].

We found some differences in opinion between current (experienced) and future (new graduates) preceptors. Many experienced preceptors expressed low comfort levels and low intention for teaching students about SUD/OUD because of self-reported inadequate training and exposure to such patients. While recent graduates were less likely to express this diffidence, some suggested that students would be better taught on addiction medicine rather than primary care rotations. This discomfort has been noted in prior studies [43, 44]. Since 13 of 15 of our participants had received MAT training, this observation suggests that MAT training alone is inadequate to address the discomfort.

An understanding of preceptor comfort level with both patient care and teaching will inform future strategies to improve and optimize student learning. Our findings suggest that SUD/OUD curricula need to be developed not only for students but also offered to both new and experienced preceptors. Such curricula should address negative bias [45] along with skills for appropriate prescribing, recognition [42] and management [46]. In our framework (Fig. 1), adding practice infrastructure support to care for patients with SUD/OUD is likely to also benefit student teaching.

Strengths of the study include diversity (MDs, PAs and NPs with varied background and practice experience) among preceptors sampled, and the inclusion of both experienced preceptors and new graduates. The research team represented expertise from clinical patient care, education, pharmacy, substance use disorder, and anthropological fields. We followed a robust coding process and achieved thematic saturation [47]. There are some study limitations. Sampling was limited to preceptors who served our institutional programs. In particular, the new preceptor sample comprised only PAs. However, there is no reason to believe that PA graduates’ opinions would differ substantially from NP or MD graduates. Differences between current and future preceptors were identified but other differences that were not identified in the present study were possible. The nature of the research was specific to PAs. However, the exclusion of other roles may have limited our findings. We acknowledge the topic remains controversial. Therefore, participants willingness to share some opinions might have been hindered despite our efforts to ensure anonymity and confidentiality.

In conclusion, our study informs efforts to better prepare preceptors to teach students about the management of SUD/OUD, by identifying barriers and facilitators to effective clinical teaching. Barriers and facilitators range from self to patient to student to systems factors. While preceptors are eager to share and transmit their knowledge and skills, they also need more local support, continuing education, and faculty development to feel as comfortable and ready to teach in this area as in other areas of chronic disease care. Future studies should aim to design, pilot, and identify best practice faculty development approaches and to solicit student input.

## Supplementary Information

**Additional file 1. **Appendix A.

## Data Availability

The data supporting the current study are protected for the privacy of our participants as outlined in the study information sheet. Deidentified data are available from the corresponding author [E.L.] on reasonable request.
