# Supplementary material for: Preceptors’ preparedness to teach about substance and opioid use disorder: a qualitative study
Source: BMC Med Educ. 2022 Dec 14;22:867. doi: 10.1186/s12909-022-03922-6 (PMC9749622; doi:10.1186/s12909-022-03922-6)
Supplement: Supplementary file 1 — Additional file 1. Appendix A. [file 12909_2022_3922_MOESM1_ESM.docx]

Appendix A.

| **Item Num.** | **Topic** | **Guide Questions/Description** | **Present Study** |
| --- | --- | --- | --- |
| **Domain 1: Research team and reflexivity** | | | |
| *Personal characteristics* | | | |
| 1 | Interviewer/facilitator | Which author/s conducted the interviews? | E.L. |
| 2 | Credentials | What were the researcher's credentials? | MSc |
| 3 | Occupation | What was their occupation at the time of the study? | Project Specialist |
| 4 | Gender | Was the researcher male or female? | Female |
| 5 | Experience and training | What experience or training did the researcher have? | Formal/didactic educational experience, 2 years of anthropological training/field work, and 3 years of real-life experience conducting interviews and focus groups |
| *Relationship with participants* | | | |
| 6 | Relationship established | Was a relationship established prior to study commencement? | Nothing beyond scheduling |
| 7 | Participant knowledge of the interviewer | What did the participants know about the researcher? | Unknown. No information provided. |
| 8 | Interviewer characteristics | What characteristics were reported about the interviewer? | Interviewer self-reported as Project Specialist. |
| **Domain 2: Study design** | | | |
| *Theoretical framework* | | | |
| 9 | Methodological orientation and Theory | What methodological orientation was stated to underpin the study? | Grounded theory |
| *Participant selection* | | | |
| 10 | Sampling | How were participants selected? | Convenience and snowball sampling |
| 11 | Method of approach | How were participants approached? | Email |
| 12 | Sample size | How many participants were in the study? | 15 |
| 13 | Non-participation | How many people refused to participate or dropped out? | 10 participants who expressed interest in participating in an interview did not reply to follow-up emails. |
| *Setting* | | | |
| 14 | Setting of data collection | Where was the data collected? | Workplace |
| 15 | Presence of non-participants | Was anyone else present besides the participants and researchers? | No |
| 16 | Description of sample | What are the important characteristics of the sample? | Current preceptors associated with the Physician Assistant program and Physician Assistant future preceptors who graduated from the program |
| *Data collection* | | | |
| 17 | Interview guide | Were questions, prompts, guides provided by the authors? Was it pilot tested? | Interviewees were not given questions prior to interview. Interview guide pre-tested prior to data collection. |
| 18 | Repeat interviews | Where repeat interviews carried out? | No |
| 19 | Audio/visual recording | Did the research use audio or visual recording to collect data? | Yes, audio recording. |
| 20 | Field notes | Were field notes made during and/or after the interview? | No |
| 21 | Duration | What was the duration of the interviews? | 35-45 minutes |
| 22 | Data saturation | Was data saturation discussed? | Yes |
| 23 | Transcripts returned | Were transcripts returned to participants for comment and/or correction? | No |
| **Domain 3: analysis and findings** | | | |
| *Data analysis* | | | |
| 24 | Number of data coders | How many data coders coded the data? | Four |
| 25 | Description of the coding | Did authors provide a description of the coding tree? | Yes, see Results section pg. 8 |
| 26 | Derivation of themes | Were themes identified in advance or derived from the data? | Derived from the data |
| 27 | Software | What software, if applicable, was used to manage the data? | No |
| 28 | Participant checking | Did participants provide feedback on the findings? | No |
| *Reporting* | | | |
| 29 | Quotations presented | Were participant quotations presented to illustrate the themes/findings? Was each quotation identified? | Yes, see Results section pg. 9-14 and Table 3. |
| 30 | Data and findings consistent | Was there consistency between the data presented and the findings? | Yes, see Discussion section pg. 15 |
| 31 | Clarity of major themes | Were major themes clearly presented in the findings? | Yes, see Results section pg. 8 and Table 3. |
| 32 | Clarity of minor themes | Is there a description of diverse cases or discussion of minor themes? | Yes, see Results section pg. 9-14 and Table 3. |
| *Adapted from: Tong A, Sainsbury P, Craig J. Consolidated criteria for reporting qualitative research (COREQ): a 32-item checklist for interviews and focus groups. International Journal for Quality in Health Care. 2007. Volume 19, Number 6: pp.349-357.* | | | |
